# A New College

**Published:** 1898-11

**Authors:** 


### A New College.

A few days ago an account of the organization of a new dental college in DesMoines, Iowa, came to hand. Judging from the statement there given it seems that extensive preparation has been made for a new institution. Thus giving us so far as we know three colleges for Iowa. Whether there is really on the part of the profession in Iowa, a demand for this third college, will probably be questioned by many. If the organization was made because of the recognized need, there ought to be no criticism, provided, it is well equipped with teachers, appliances, buildings, etc., for the work.

About this we are not sufficiently advised to give an opinion. If it is to do thorough work it may be welcomed into the sisterhood of colleges. If it was put in operation, in the main, to give place to aspirants, it would be better that the effort had not been made; of course the outcome will be revealed in the near future.

